# Multi-Link Operation with Enhanced Synchronous Channel Access in IEEE 802.11be Wireless LANs: Coexistence Issue and Solutions

**DOI:** 10.3390/s21237974

**Published:** 2021-11-29

**Authors:** Wisnu Murti, Ji-Hoon Yun

**Affiliations:** Department of Electrical and Information Engineering, Seoul National University of Science and Technology, Seoul 01811, Korea; wisnumurti@seoultech.ac.kr

**Keywords:** multi-link operation, IEEE 802.11be, Extremely High Throughput, channel access

## Abstract

Multi-link operation is a new feature of IEEE 802.11be Extremely High Throughput (EHT) that enables the utilization of multiple links using individual frequency channels to transmit and receive between EHT devices. This paper aims to illustrate enhanced multi-link channel access schemes, identify the associated coexistence challenge, and propose solutions. First, we describe the multi-link operation of IEEE 802.11be and how the asynchronous and synchronous channel access schemes facilitate multi-link utilization. Next, we describe the design variants of the synchronous channel access scheme and demonstrate the associated coexistence challenge. Subsequently, we propose four features to address this challenge by assigning penalties to multi-link devices (repicking a backoff count, doubling the contention window size, switching to another contention window set, and compensating the backoff count) as well as five coexistence solutions derived from combinations of these features. Comparative simulation results are provided and analyzed for dense single-spot and indoor random deployment scenarios, demonstrating that the throughput and latency gains of multi-link operation differ between schemes. At the same time, we investigate the coexistence performance of multi-link operation with and without the capability of simultaneous transmission and reception and demonstrate that the proposed solutions mitigate the coexistence problem. In particular, compensating the backoff count achieves the highest coexistence performance among the proposed solutions, with a marginal throughput decrease of multi-link devices. A metric for evaluating both the throughput and latency gains and the coexistence performance of a multi-link channel access scheme using a single value is also proposed.

## 1. Introduction

Mobile data traffic is expected to grow at a compound annual growth rate (CAGR) of 46% by 2022 [1]. One of the main solutions to meet such increasing demands for bandwidth has long been leveraging Wi-Fi networks. 59% of total mobile data traffic is expected to be delivered through Wi-Fi networks by 2022 [1]. For the continuing evolution of Wi-Fi networks [2], the next generation of IEEE 802.11 wireless local area network (WLAN) technology, named *Extremely High Throughput* (EHT), is under development by the 802.11be task group of IEEE (TGbe) [3,4], and is expected to be adopted by the Wi-Fi Alliance as Wi-Fi 7. EHT focuses on achieving an extreme increase in the peak throughput up to 30 Gbps, which is three times higher than that of Wi-Fi 6, while simultaneously reducing latency [5,6,7,8,9]. Mobile gaming and wireless virtual reality (VR) are two main use cases that are encouraging the development of EHT towards high peak throughput and low latency, whereas Wi-Fi 6 (also known as High Efficiency Wi-Fi or 802.11ax) was developed to ensure a stable user experience in dense deployment environments.

A new feature of the medium access control (MAC) layer for the realization of EHT is *multi-link operation*. While physical-layer enhancements, including new bands, wider bandwidths, and higher spatial and modulation orders, are also to be adopted, as was done for prior generations, multi-link operation is a new approach being attempted for the first time in EHT. The idea is to enable a pair of devices to use multiple wireless links in different bands simultaneously for transmission and reception. The main benefit of this approach is the simultaneous exploitation of multiple bands at a lower hardware cost than that of a single multiband radio. This multi-link operation capability is different from the multiband support offered by the access points (APs) currently available on the market in that multi-link operation allows the concurrent use of multiple links at client devices and thus also enhances the throughput of a single data session (Multiband APs allow client devices to connect using only one band at a time).

The main challenges of multi-link operation are (1) the design of channel access schemes with and without the presence of radio frequency (RF) power leakage between links, and (2) the coexistence of devices using multi-link channel access schemes with legacy devices. A straightforward approach to channel access in multi-link operation is to run individual links with independent channel access processes, as in the conventional case; however, this is only possible when no RF power leakage exists between the links. If RF power leakage is present between the links due to imperfect RF shielding, filter artifacts, etc., then synchronous channel access is preferred. Moreover, multi-link devices using these channel access schemes must coexist well with legacy devices when occupying the shared medium.

There have been some research attempts toward multi-link aggregation in the unlicensed spectrum. Glia [10] is a software implementation combining orthogonal frequency channels of multiple center frequency bandwidths and can achieve a high data rate through semisynchronous channel access. E-MICE [11] exploits multiple Wi-Fi radio links in individual bands concurrently with the aid of either link-layer aggregation or the Multipath Transmission Control Protocol (MPTCP). It is equipped with an algorithm to dynamically turn on/off links and switch between them to minimize energy consumption with little performance degradation. Naribole et al. [12] have developed a constraint-aware aligned downlink ending protocol for simultaneous downlink transmissions over multiple channels for devices with no ability to simultaneously transmit and receive. Listen-before-talk schemes (LBT) for multicarrier aggregation of Long-Term Evolution with License-Assisted Access (LTE-LAA; LTE for unlicensed spectrum) [13,14] have been studied, and enhanced schemes for use in the presence of RF power leakage were proposed in [15,16,17]. As proposed by the above-mentioned works, multi-link operation requires a new channel access scheme to better exploit multi-link transmission opportunities. However, none of these prior research works focused on the issue of coexistence with legacy single-link Wi-Fi devices for multi-link channel access schemes. Exploring the coexistence problem and designing solutions within the framework of EHT will be essential to facilitate the wide and fast penetration of the new approach.

This paper aims to illustrate enhanced multi-link channel access schemes for IEEE 802.11be EHT, identify the associated coexistence challenge, and propose solutions applicable to cases both with and without the capability of simultaneous transmission and reception. First, we describe the concept, architecture, and asynchronous and synchronous channel access schemes of multi-link operation, along with the RF power leakage problem. Next, we describe the design variants of the synchronous channel access scheme and demonstrate the coexistence challenge of the enhanced scheme. Subsequently, we propose four features to mitigate this challenge by assigning penalties to multi-link devices: repicking a backoff count, doubling the contention window size, switching to another contention window set, and compensating the backoff count. Then, five coexistence solutions are derived from combinations of these features. Comparative evaluation results are provided and analyzed for dense single-spot and indoor random deployment scenarios, demonstrating that the throughput and latency gains of multi-link operation differ between schemes. We also investigate the coexistence performance of multi-link operation with and without the capability of simultaneous transmission and reception and demonstrate that the proposed solutions mitigate the coexistence problem to different degrees. In particular, compensating the backoff count achieves the highest coexistence performance among the proposed solutions, with a marginal throughput decrease of multi-link devices. A metric for evaluating both the throughput and latency gains and the coexistence performance of a multi-link channel access scheme using a single value is also proposed.

In summary, the main contributions of our work are listed as follows:

- Identification of the issue concerning the coexistence of an enhanced synchronous multi-link channel access scheme with legacy devices, which has not been explored yet in the literature.
- Design of features to assign a penalty to a backoff process and coexistence solutions derived from various feature combinations.
- Comprehensive simulation to illustrate the throughput and latency gains and coexistence performance of various multi-link channel access schemes including the proposed solutions when multi-link devices coexist with legacy devices.
- Design of a new metric for evaluating both the throughput and latency gains and the coexistence performance of multi-link operation using a single value.

The rest of this paper is organized as follows. Section 2 explains the details of multi-link operation. Section 3 describes the variants of the synchronous multi-link channel access scheme and the coexistence issue. Section 4 details the proposed solutions, and Section 5 presents simulation-based evaluations of the throughput and latency gains along with the coexistence performance of various multi-link channel access schemes in the presence of legacy devices. Finally, Section 6 concludes the paper.

## 2. Overview of Multi-Link Operation in IEEE 802.11be WLANs

Multi-link operation (MLO) is the concurrent utilization of multiple radio links of different frequency channels/bands by an AP, a client, or both. It is a MAC-layer solution for concurrently using multiple links and thus exposes a single MAC service access point (SAP) to the upper sublayer of the data link layer, i.e., the logical link control (LLC) layer. Therefore, existing user services and higher-layer protocols need not expend any effort to enjoy its benefits.

The motivation for MLO is to increase throughput by aggregating multiple bandwidths across bands at a low hardware cost. Ideally, the maximum achievable throughput of MLO is the sum of the achievable throughput for each link. For example, if each of *n* links can achieve identical throughput, their MLO should ideally achieve n× throughput.

This throughput increase enabled by MLO comes from the use of a wider bandwidth through the aggregation of multiple links. MLO has several advantages over conventional single-link operation (SLO) using a wide bandwidth. First, it allows the aggregation of available bandwidths across bands, such as 2.4, 5, 6 and even 60 GHz, at an affordable hardware cost by reusing most of the existing physical (PHY)-layer and lower-MAC (LMAC) functionalities while reinventing the upper-MAC (UMAC) component only. Second, it has the opportunity to run multiple channel access processes in individual links thanks to multiple RF chains. This is beneficial in exploiting each link’s bandwidth in a manner that is more adaptive to its conditions, such as contention intensity. In contrast, the conventional wideband operation of IEEE 802.11 runs a single channel access process in a primary channel only, and thus significantly depends on the conditions of this single channel; for example, if the primary channel is crowded, fewer transmission opportunities are available for wideband operation regardless of the conditions of the other channels.

### 2.1. Multi-Link Device and Network Architecture

**Figure 1:**
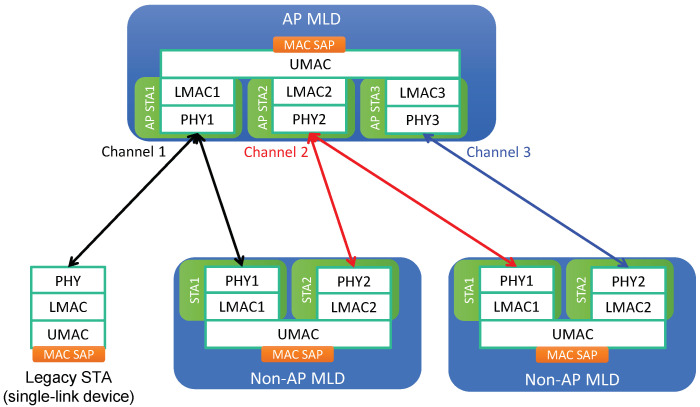
Multi-link system architecture.

The system architecture for MLO is illustrated in Figure 1. An EHT device that is capable of MLO is called a *multi-link device* (MLD), while a legacy device supporting SLO only is called a *single-link device* (SLD). Each link of an MLD is regarded as a conventional single-link station (STA), and an MLD is considered to be a device affiliated with more than one STA. In contrast, an SLD (legacy device) is affiliated with only a single STA. Thus, we use the terms “legacy device” and “legacy STA” interchangeably. MLDs can be further classified into two types based on the type of the affiliated STAs:

- AP multi-link device (AP MLD): each of the affiliated STAs is an AP.
- Non-AP multi-link device (non-AP MLD): each of the affiliated STAs is a non-AP (client) STA.

Each affiliated STA of an MLD is composed of PHY and LMAC components. On top of the set of affiliated STAs, an MLD has one UMAC component to aggregate its set of affiliated STAs and provide LLC with a single MAC SAP. An AP STA and any non-AP STAs associated with it form a basic service set (BSS). As illustrated in Figure 1, an AP MLD can manage non-AP MLDs through the establishment of different links. A non-AP MLD can be linked to multiple non-AP STAs using individual channels, each of which is associated with an AP STA of the AP MLD.

### 2.2. Multi-Link Transmission and Reception

IEEE 802.11 defines a traffic identifier (TID) to classify a packet for differentiated service. The TID is represented as a four-bit number (0–7) identifying the desired quality of service (QoS) for the traffic. In MLO, the TID is used to determine which link(s) to use for traffic with a specific QoS. If a specific TID is mapped to a set of links, then any link within that set can be used to transmit data frames from that TID. By default, after multi-link setup, all TIDs are mapped to all established links. However, the TID-to-link mapping can specify either the same or different link sets for each TID. After multi-link setup, the TID-to-link mapping can be updated through negotiation.

#### 2.2.1. One-to-One TID-to-Link Mapping (Flow-Level Aggregation)

In this mapping strategy, each TID is mapped to a single link, meaning that traffic with a given TID will be transmitted using the associated dedicated link only. Distributing individual traffic flows defined by different TIDs to different links in this way will lead to increased throughput and decreased latency. However, since TID-to-link mapping updates may not be frequent, the effect of the throughput increase achieved with this strategy is somewhat limited; even if the amount of traffic associated with a particular TID is large and the corresponding link queue becomes congested, other links cannot be used for this TID. For the same reason, however, this mapping strategy is beneficial for high-priority TIDs because it physically isolates the associated link resources from other lower-priority TIDs.

#### 2.2.2. One-to-Many TID-to-Link Mapping (Packet-Level Aggregation)

**Figure 2:**
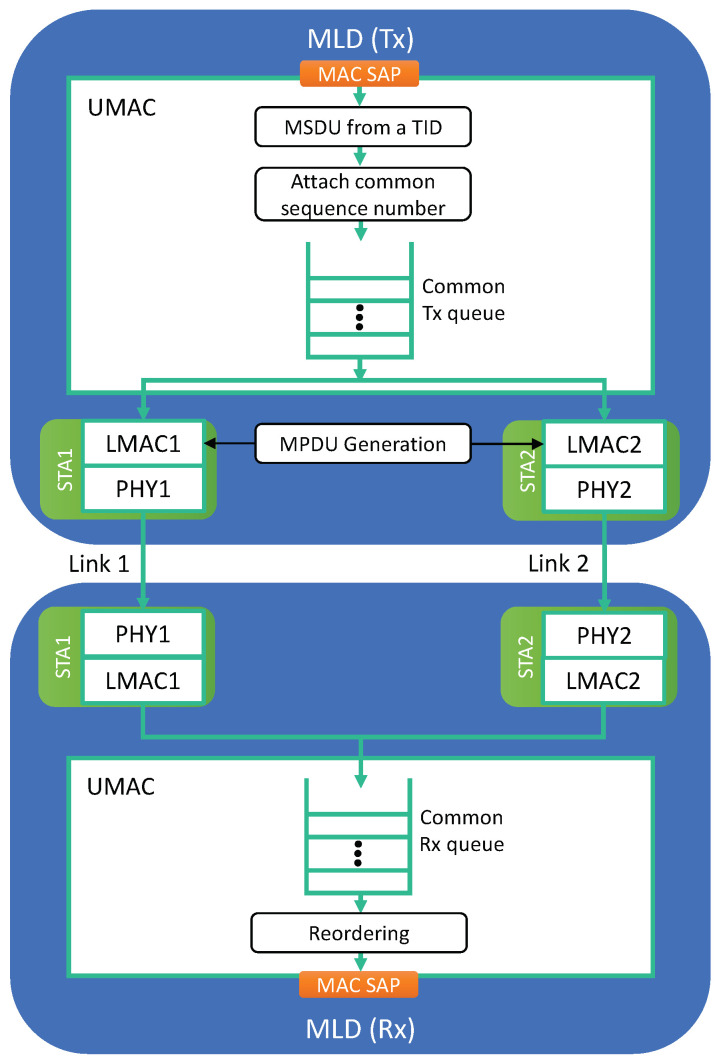
Common UMAC and queuing architecture for packet-level link aggregation.

To fully exploit the throughput increase and latency decrease effects of MLO, a TID can be mapped to a set of multiple links, allowing traffic with that TID to be transmitted using any link in this set, as illustrated in Figure 2. In this way, a higher rate of traffic with that TID can be handled, and a congested queue for that TID will be flushed faster. The UMAC on the transmitter side plays a role in distributing traffic of each TID to its mapped links (LMACs) at the packet level.

However, such a strategy is inherently accompanied by the reordering of packets; thus, the UMAC on the receiver side must buffer the received traffic and pass packets in sequence only to the LLC layer. Therefore, received packets may wait in the receiver buffer until they can be arranged in sequence. To achieve this, multiple links for same-TID packets must use a common sequence number space shared across multiple links.

## 3. Multi-Link Channel Access Schemes and Coexistence Issue

For multi-link channel access, multiple operation options are considered. The usability of these options is related to the simultaneous transmission and reception (STR) capability of a device. In what follows, we first explain the STR capability, describe two basic multi-link channel access schemes, and present variants for enhancement along with the coexistence issue.

### 3.1. STR Capability

**Figure 3:**
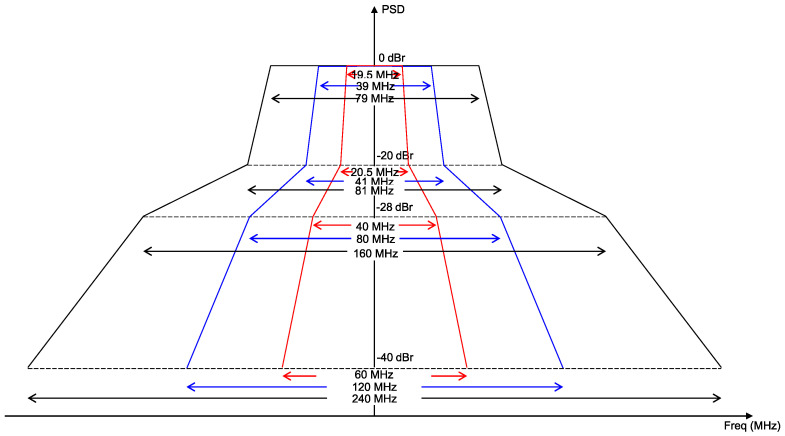
Spectral mask requirements of an IEEE 802.11 transmitter.

Since multiple radio interfaces exist within a single MLD, near-band in-device interference may arise. Figure 3 shows the transmit spectral mask requirements of an IEEE 802.11 transmitter in the 20, 40, and 80 MHz bandwidths [18]. As shown in this figure, for a transmit power of 1 mW, the maximum allowable power leakage to other channels ranges from −20 dBm (for neighboring channels) to −40 dBm (for other channels); for a typical level of transmit power (e.g., 20 dBm), this range will be higher. These RF power leakage levels are higher than the energy detection (ED) threshold of IEEE 802.11, which is −62 dBm. This may cause other links that are contending in other channels to falsely detect such leakage as a busy medium.

If an MLD is equipped with high-fidelity filters and shielding, this RF power leakage problem can be avoided, thus enabling STR. However, if the level of power leakage to the nontransmitting links of an MLD ruins either their clear channel assessment (CCA), signal reception, or both (We consider the case in which the power leakage of a transmitting link ruins both the CCA and reception of other links in an MLD), it does not have the STR capability. Depending on whether they possess the STR capability, MLDs can be classified into the following two types:

- STR MLD: A transmission on one link does not affect the operations of frame reception and CCA on other links. Therefore, individual links can operate independently of each other.
- Non-STR MLD: Operation on one link is restricted by operation on another link; a transmission on one link is not allowed if it will cause reception interruption on another link, or a reception or CCA on one link is not allowed if a transmission is ongoing on another link.

In general, an AP MLD should be capable of STR, while different non-AP MLDs may have different STR capabilities. Therefore, IEEE 802.11be WLANs support the following cases: STR AP MLDs with STR non-AP MLDs and non-STR non-AP MLDs. It is still important to provide non-STR MLDs with throughput enhancement and latency reduction close to those of STR MLDs.

### 3.2. Multi-Link Channel Access Schemes of EHT

**Figure 4:**
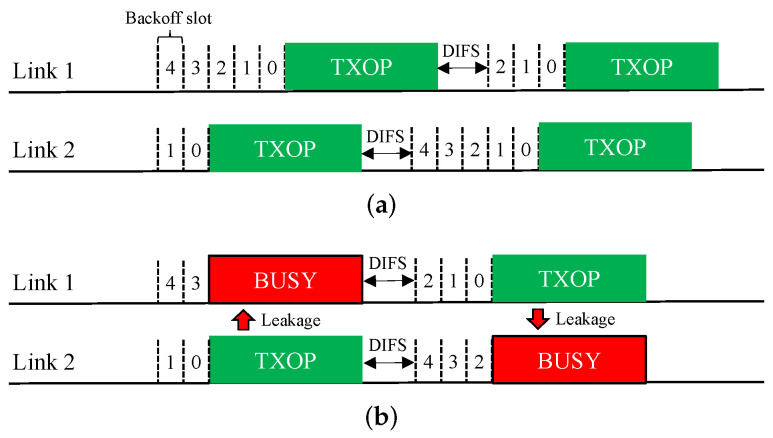
Asynchronous operation (Async) scheme for multi-link channel access. (**a**) STR MLD. (**b**) Non-STR MLD.

**Asynchronous Operation (Async)**. The MLO functionality of EHT allows an asynchronous multi-link channel access scheme in which each of the STAs belonging to an MLD performs a channel access process (backoff procedure, BO) over its own link independently. Therefore, as illustrated in Figure 4a, the BOs of different links may finish at different times, and the transmissions over these links will be asynchronous. In addition, downlink and uplink frames can be transmitted simultaneously over multiple links.

This Async scheme is ideal for STR MLDs. However, if it is applied to a non-STR MLD, as shown in Figure 4b, a transmission on a link will appear as interference beyond the ED threshold on other links, thus causing them to sense a busy medium and freeze their BOs until the transmission ends. Therefore, for a non-STR MLD, Async significantly degrades the ability to utilize multiple links.

**Figure 5:**
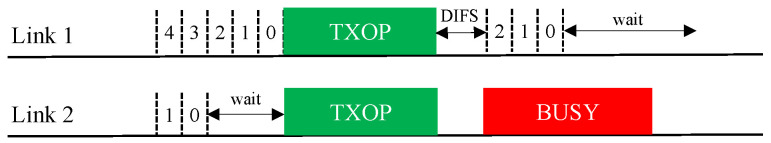
Synchronous operation (Sync) scheme for multi-link channel access.

**Synchronous Operation (Sync)**. The synchronous multi-link channel access scheme of EHT is illustrated in Figure 5. In this scheme, individual BOs are performed on all links. If a link finishes its BO early, it waits until the other links have also finished their BOs. If the channel of a link that has been waiting after finishing its BO becomes busy, the link must rerun its BO (the contention window (CW) remains unchanged).

The Sync scheme is specifically designed for non-STR MLDs but is also applicable to STR MLDs. Because it only allows simultaneous transmission initiation on all links, it is unaffected by RF power leakage between links and thus operates similarly in STR and non-STR MLDs. However, it may not utilize multiple channels as optimally as Async since all links must have backoff counts of zero when their individual channels are idle.

### 3.3. Design Variants of Synchronous Channel Access Operation

**Figure 6:**
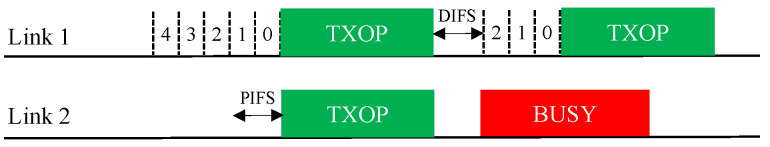
Sync-PL operation.

**Figure 7:**
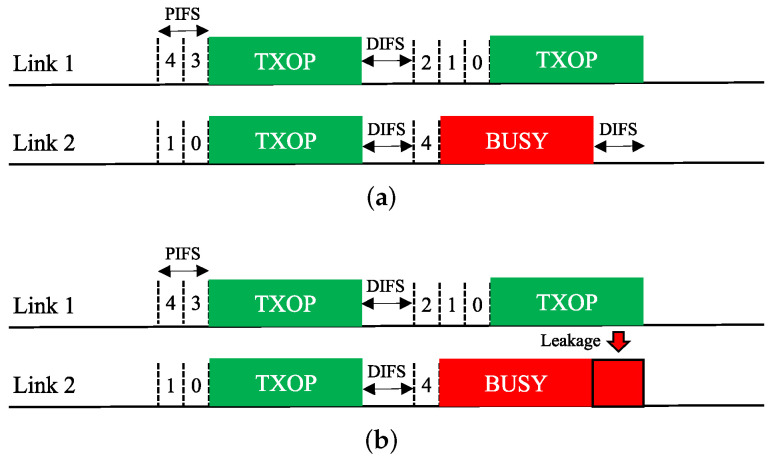
Sync-FT operation. (**a**) STR MLD, (**b**) Non-STR MLD.

We consider two design variants of the Sync scheme for better exploitation of multi-link transmission opportunities:

- *Sync-PL*: This scheme resembles the conventional wideband operation of IEEE 802.11 [6,18], as illustrated in Figure 6. In this operation scheme, an MLD runs a single BO in a primary link (PL) only. When the backoff count of the primary link is about to reach zero (within one point coordination function interframe space (PIFS) before the backoff completion time), the MLD performs a short CCA for a PIFS on each of the other links to check the availability of each. At the time of backoff completion, the MLD transmits frames using the primary link and the other link(s) sensed to be idle such that the transmissions on the used links will finish at the same time. If no other link is available at this time, the MLD uses the primary link only. In this manner, the transmit opportunities (TXOPs) of different links are synchronized with each other.
- *Sync-FT*: This scheme is a hybrid of the Sync and Async schemes for faster transmission (FT) when a multi-link transmission opportunity is available, as illustrated in Figure 7, with the aim of providing both STR and non-STR MLDs with high multi-link utilization [19,20,21]. As in Async, the links of an MLD are allowed to run individual BOs. When some of the links finish their BOs earlier than the others, the MLD performs a short CCA on each of the other links and begins to transmit on the set of links that are sensed to be idle, as in Sync, freezing the backoff counts of the links. Once the links complete transmission, they resume their individual BOs; those that have completed their BOs at the time of transmission pick a new backoff count, while the others resume their BOs with their remaining (frozen) backoff counts.
  This operation scheme lessens the failure of link utilization in non-STR MLDs due to RF power leakage, thus enhancing their multi-link utilization. As illustrated in Figure 7, Sync-FT is applicable to both STR and non-STR MLDs but with a subtle difference in behavior.

### 3.4. Coexistence Issue for Sync-FT Operation

**Figure 8:**
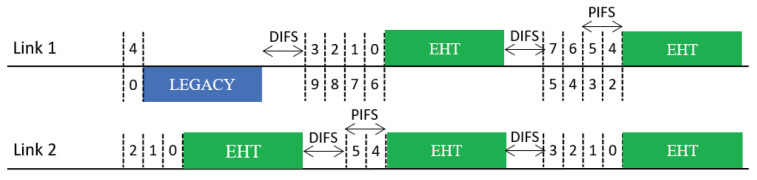
Issue concerning the coexistence of EHT MLDs with legacy devices.

In practical WLANs, legacy SLDs may coexist with EHT MLDs. Therefore, a coexistence issue arises between them. An illustration of such a coexistence scenario is given in Figure 8. In the scenario depicted in this figure, a legacy device starts a BO with the same backoff count as an MLD under the Sync-FT scheme. During the illustrated operation period, the legacy device obtains a single TXOP on Link 1, while the MLD obtains two TXOPs. This is because the Sync-FT scheme enables the MLD to transmit on a link before backoff completion on that link; we call such a link a *free-riding link* in this paper. A free-riding link resumes its ongoing BO after transmission is complete, using the count value frozen immediately before the transmission. This behavior prioritizes channel access for the MLD and provides it with more TXOPs, resulting in long and frequent wait times for the legacy device. This behavior can also be interpreted as the free-riding link starting a new BO with a relatively deflated backoff count.

## 4. Coexistence Solutions for Sync-FT Operation

**Figure 9:**
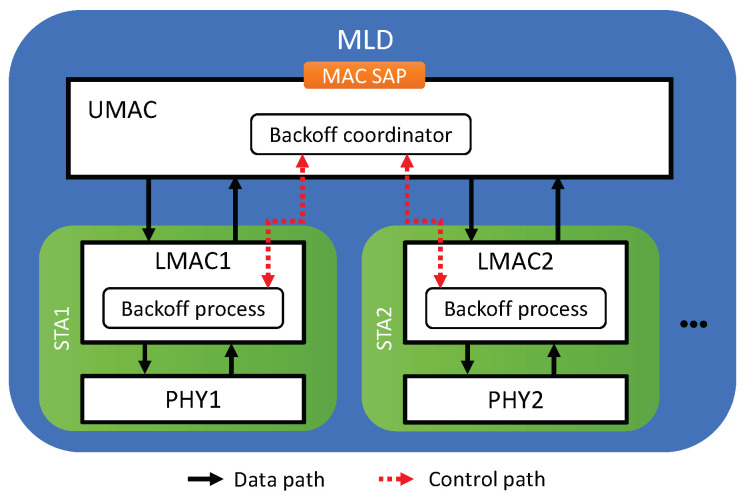
Proposed architecture of an MLD.

**Figure 10:**
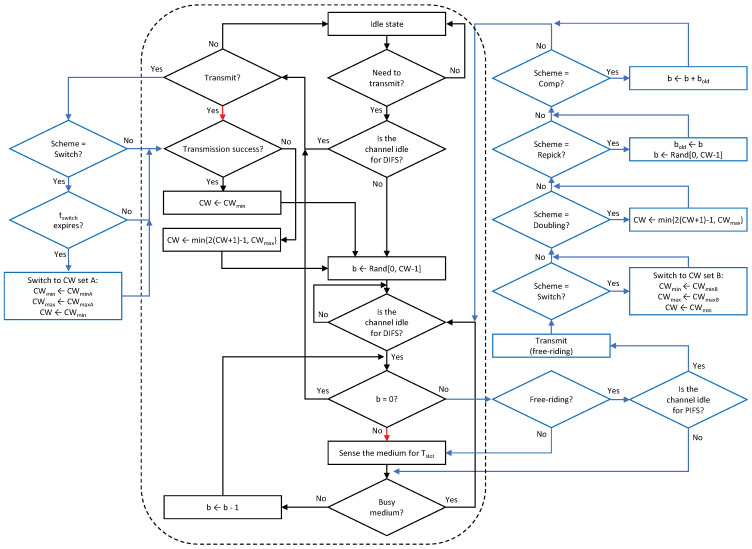
Flowchart of the channel access procedure of a link under the Sync scheme with the proposed solutions (black-outlined boxes + black and red arrows: legacy operations, all boxes + black and blue arrows (excluding red ones): new operations).

In what follows, we describe the details of the proposed solutions. The approach of the proposed solutions is to assign a free-riding link a penalty for its subsequent channel access following a free-ride transmission so that each link of an MLD will experience a similar amount of backoff time as a legacy device does in the same channel. We propose four features for assigning penalties to free-riding links. Then, various combinations of these features are considered as solutions, as listed in Table 1. The proposed architecture of an MLD is depicted in Figure 9 where the proposed solutions are implemented by the interaction between the backoff processes of individual links through the backoff coordinator of the UMAC. The entire procedure for channel access by a link is illustrated in Figure 10 and its pseudocode is given in Algorithm 1, where a new flag variable named Scheme indicates the features to be activated.

### 4.1. Repicking a Backoff Count

**Figure 11:**
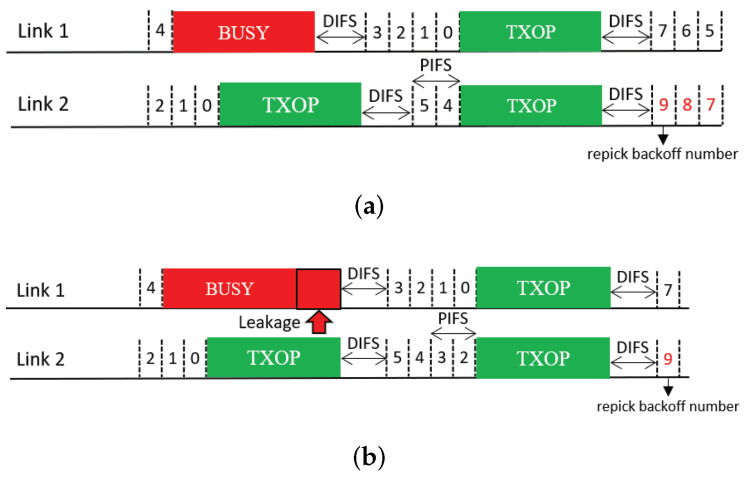
Repicking a backoff count (Repick). (**a**) STR MLD, (**b**) Non-STR MLD.

This feature allows a free-riding link to repick a new backoff count and restart a BO based on this new count immediately after a free-ride transmission (Figure 11); thus, we name it *Repick*. Since the link may have performed a BO before the free-ride transmission and reduced the backoff count by some amount, allowing it to repick a new backoff count will increase its backoff time on average. In the example shown in the figure, the free-riding link (Link 2) with Repick starts a new BO with a backoff count of nine. Without this feature, the link would use the previous count of three (STR case) or one (non-STR case). The newly repicked count value is not affected by whether the free-riding transmission was successful. It follows the current CW value of the link.

### 4.2. Doubling the CW

**Figure 12:**
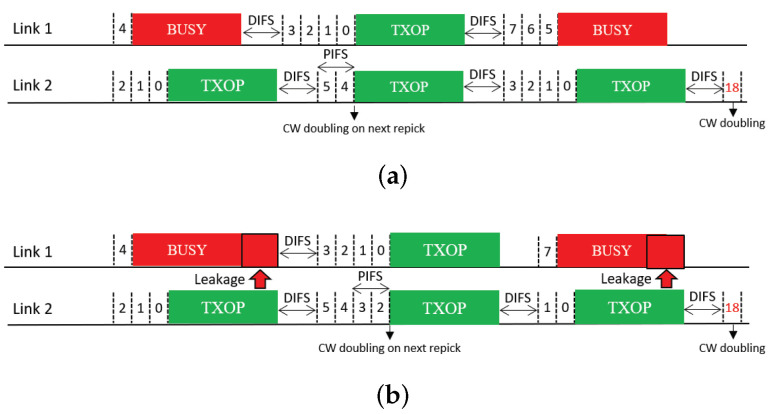
CW doubling (Double). (**a**) STR MLD, (**b**) Non-STR MLD.

**Figure 13:**
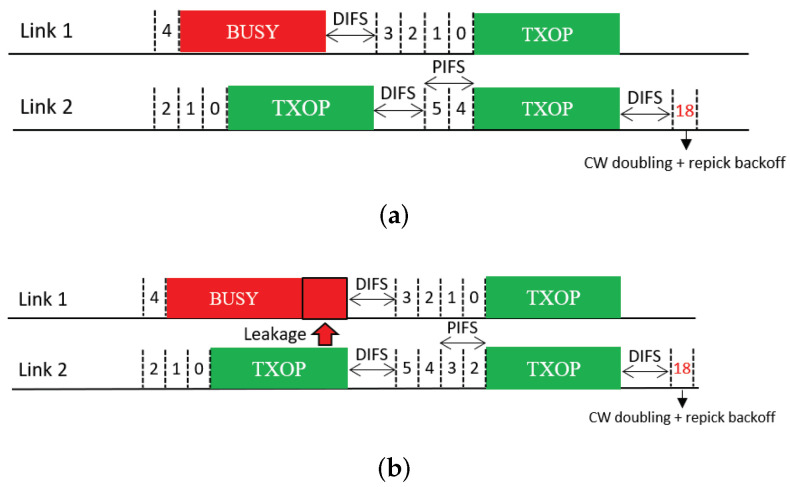
CW doubling and repicking a backoff count (Double + Repick). (**a**) STR MLD, (**b**) Non-STR MLD.

The next feature uses CW doubling as a penalty. Upon completion of a free-riding transmission, the CW of the corresponding link is doubled; consequently, the next backoff count to be picked for this link will have a higher value on average. We propose two solutions that use this feature, which we call *Double* and *Double + Repick*, respectively. Double allows an STA to continue its BO with its remaining backoff count, as illustrated in Figure 12. Once the STA transmits upon completion of the BO, its CW is then doubled as a penalty regardless of the transmission result, and the next backoff count is picked. As illustrated in Figure 13, under Double + Repick, an STA doubles its CW and immediately repicks a new backoff count after a free-riding transmission finishes. That is, the difference between the two solutions is the use of the feature Repick after the doubling of the CW. Therefore, Double+Repick will assign a larger penalty to a free-riding link.

### 4.3. Switching to Another CW Set

**Figure 14:**
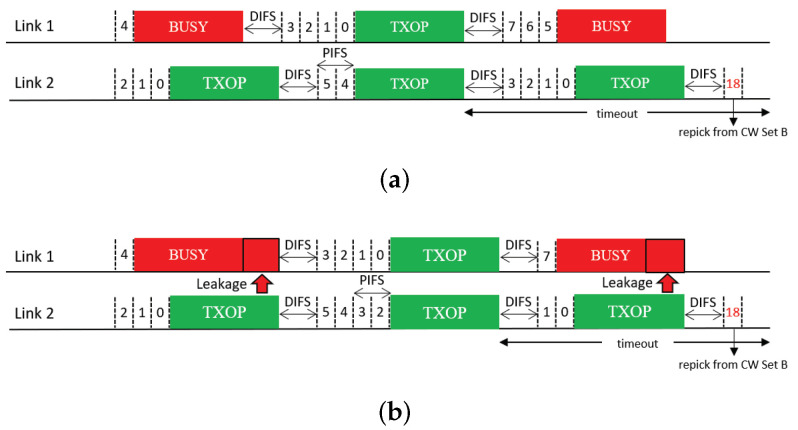
Switching to another CW set (Switch). (**a**) STR MLD, (**b**) Non-STR MLD.

This feature is inspired by the multi-user mode of enhanced distributed channel access (MU-EDCA) in 802.11ax [18], which penalizes an STA for channel access after it is given a channel access opportunity by a trigger-based transmission. The AP of a BSS assigns a pair of CW sets, denoted by set A and set B, to its associated STAs, where set B has CW minimum (CWmin) and maximum (CWmax) values that are the same as or higher than those of set A. The STAs perform their BOs based on CW set A by default. After an STA is given a channel access opportunity by a trigger-based transmission, it switches to CW set B. A similar approach can be adopted for MLO, as illustrated in Figure 14. We refer to the corresponding solution as *Switch*. After a link is given a channel access opportunity by free riding, we switch the link to CW set B so that it has to wait longer on average for channel access due to its own BO completion. A timer ($t_{switch}$) is started when the link switches to CW set B. When the timer expires, the link switches back to CW set A. Switching from one set to another is not affected by the transmission result, and the CW value is reset to the CWmin of the current set.

### 4.4. Compensation of the Backoff Count

**Figure 15:**
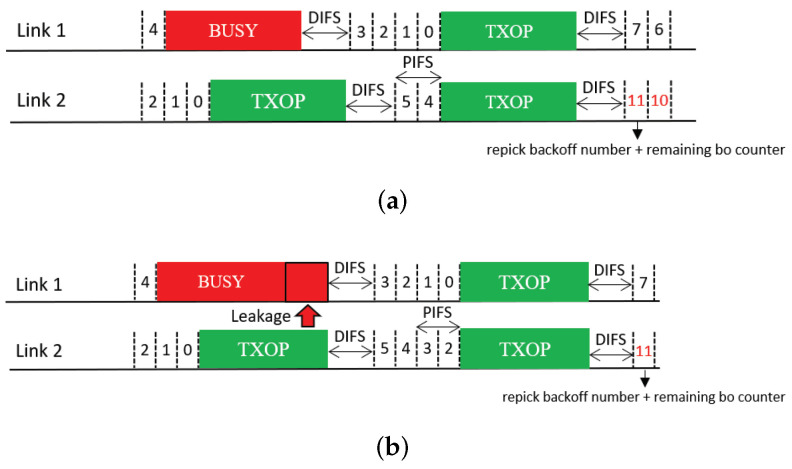
Compensation after repicking a backoff count (Repick + Comp). (**a**) STR MLD, (**b**) Non-STR MLD.

The last feature compensates the backoff count of a free-riding link by a certain amount such that it will ultimately go through the full backoff time length on average. The operation of the solution that utilizes this feature is illustrated in Figure 15. This feature requires an STA to repick a new backoff count value to be added to the current count. Thus, we refer to the solution that includes this feature as Repick + Comp. In the example shown in the figure, the MLD repicks a backoff count value of eight (STR case) or ten (non-STR case) for Link 2 (free-riding link). After the free-riding transmission finishes, the link stores its current backoff count value as $b_{old}$ and repicks a new backoff count *b*. Then, it performs a new BO with an initial count of $b+b_{old}$. In this example, the backoff count of Link 2 becomes eleven after compensation instead of three (STR case) or one (non-STR case).
**Algorithm 1** Proposed multi-link channel access procedure of an STA.1:**while** the STA has a packet to transmit **do**2: b←Rand[0,CW−1] # Pick a new backoff count value3:   **while** b!=0 **do**4:  **if** Other STA has completed a backoff process and is about to transmit a packet **then**5:   **if** the channel is idle for PIFS **then**6:    Transmit the packet in the given channel  7:    **if** the MLD uses the Switch Scheme **then**8:     $\left(CW_{min},CW_{max},CW\right)\leftarrow \left(CW_{minB},CW_{maxB},CW_{min}\right)$  9:     Start a timer with an expiration time of $t_{switch}$  10:    **end if** 11:    **if** the MLD uses the Doubling Scheme **then**12:     $CW\leftarrow \min(2\left(CW+1\right)-1,CW_{max})$  13:    **end if** 14:    **if** the MLD uses the Repick Scheme **then**15:     $b_{old}\leftarrow b$, b←Rand[0,CW−1] # Repick a new backoff count value  16:    **end if** 17:    **if** the MLD uses the Compensation Scheme **then**18:     $b\leftarrow b+b_{old}$ # Add the remaining backoff count to the current value  19:    **end if** 20:   **end if** 21:  **end if** 22:  **while** the channel is sensed busy **do**23:   Sense the channel for DIFS  24:  **end while** 25:  b←b−1 for every idle $T_{slot}$ duration  26: **end while** 27: Transmit the packet in the given channel  28: **if** the MLD uses the Switch Scheme $t_{switch}$ has expired **then**29:  $\left(CW_{min},CW_{max},CW\right)\leftarrow \left(CW_{minA},CW_{maxA},CW_{min}\right)$  30: **end if** 31: **if** the transmission succeeds **then**32:  $CW\leftarrow CW_{min}$  33: **else**34:  $CW\leftarrow \min(2\left(CW+1\right)-1,CW_{max})$  35: **end if** 36:**end while** 

## 5. Performance Evaluation

**Figure 16:**
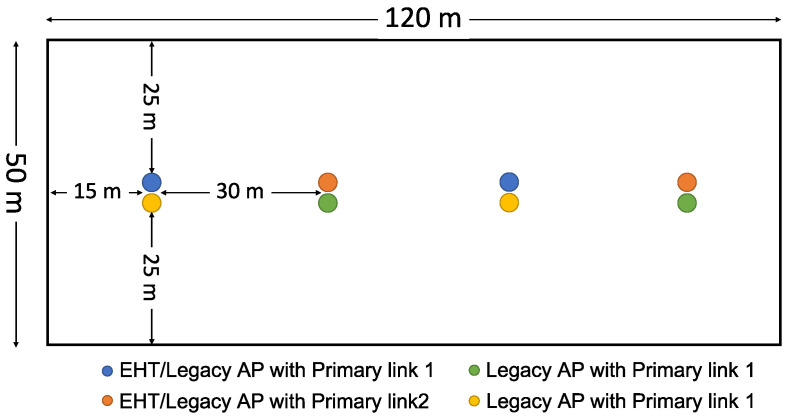
Indoor random deployment scenario.

We evaluate and compare the performance gain and coexistence performance of MLO under various channel access schemes through simulation (We used a Matlab-based in-house simulator). In addition to the two basic channel access schemes of EHT MLO, i.e., Async and Sync, we also evaluate Sync-PL and Sync-FT as well as the coexistence solutions for Sync-FT described in the previous section. In this evaluation, we consider two deployment scenarios:

- *Single-spot deployment*: All APs and devices are deployed at a single spot. APs/devices of the same type have the same modulation and coding scheme (MCS). This deployment scenario enables us to clearly observe and analyze the coexistence performance.
- *Indoor random deployment*: Following the 3rd Generation Partnership Project (3GPP)’s indoor evaluation scenario for coexistence in unlicensed spectrum [22], the APs are evenly placed, and devices with link adaptation are randomly distributed to allow the coexistence performance to be investigated under more realistic conditions. The deployment area considered in this scenario and the placement of the APs are shown in Figure 16.

**Figure 17:**
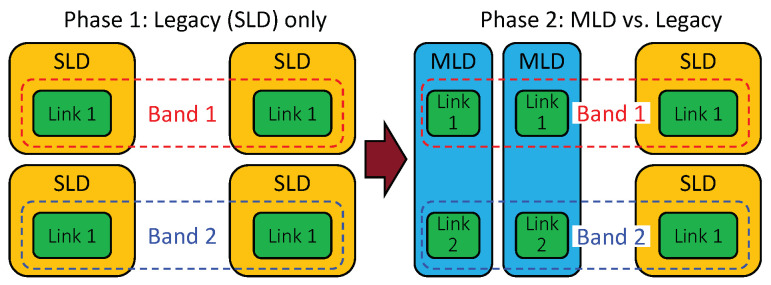
Coexistence evaluation scenario.

The coexistence performance of MLO is investigated by comparing the throughput and latency performance of legacy devices between the cases without and with MLDs. To this end, two phases of simulation are performed, as illustrated in Figure 17 and described below:

- (Phase 1) Legacy devices only: Only legacy devices exist in the simulated environment.
- (Phase 2) MLD vs. legacy devices: Half of the legacy devices from the first phase are replaced with MLDs.

In the transition from Phase 1 to 2, one legacy device is replaced with one MLD. The purpose is to evaluate the coexistence performance of MLO when the number of coexisting devices remains unchanged. Phase 1 reflects the baseline performance of the conventional single-link channel access scheme. Phase 2 reveals, compared with the performance in Phase 1, how much of a performance gain MLO achieves and how much of a performance degradation the legacy devices suffer. As the performance of the legacy devices in Phase 2 decreases relative to that in Phase 1, the MLDs coexist more poorly with the legacy devices.

The simulation parameters are listed in Table 2. Each MLD has two links working in individual channels. The cases of MLDs both with and without the STR capability are considered. A transmission frame is an aggregation of up to 64 MAC protocol data units (MPDUs), where each MPDU is 1500 bytes long. We consider full-buffer traffic conditions. For the coexistence solution of CW set switching, CW set A has a CWmin of 16 and a CWmax of 1024, while CW set B has a CWmin of 32 and a CWmax of 2048. The timeout time for switching ($t_{switch}$) is set to 100 ms. All simulation results are obtained by averaging over 5 runs; in each run, 50 s of time are simulated.

### 5.1. Single-Spot Deployment Scenario

In this deployment scenario, we first investigate the throughput gain of MLO. Then, we present the coexistence performance and evaluate it using a new metric.

#### 5.1.1. Throughput Gain of MLO

First, we consider one MLD deployed with various numbers of overlapping BSSs (OBSSs) with legacy SLDs. The MLD is contending on both link 1 and link 2. OBSSs are added to each of the links at the same time, i.e., one OBSS means one legacy BSS on link 1 and link 2. The performance gain of MLO is evaluated in terms of throughput enhancement.

**Figure 18:**
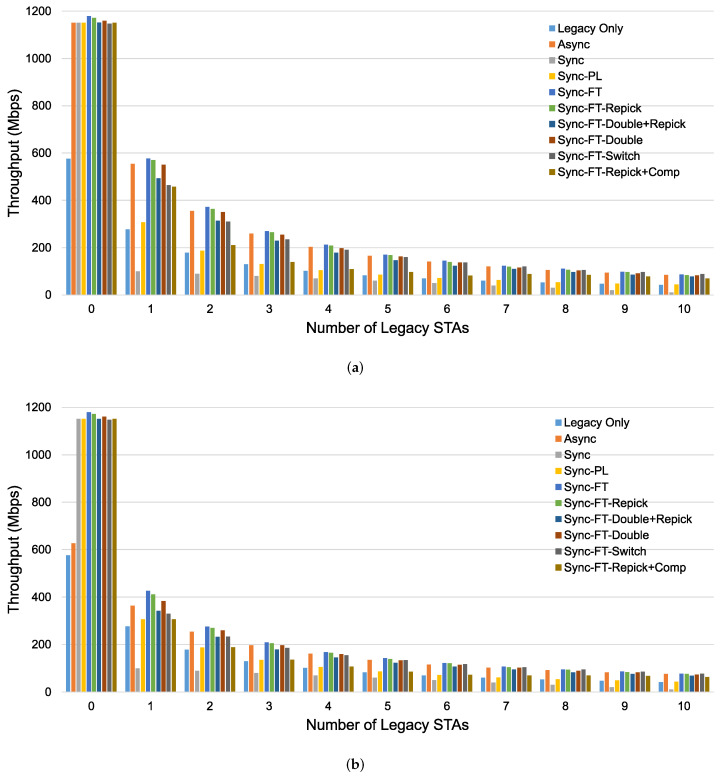
Throughput performance of an MLD network with and without the STR capability when a specified number of legacy devices coexist in the single-spot deployment scenario. (**a**) With STR capability, (**b**) Without STR capability.

The throughput performance results of MLO using different channel access schemes with coexisting legacy devices are presented in Figure 18. In the case with the STR capability, as shown in Figure 18a, the throughput of MLO is approximately double that of SLO (the legacy-only case) when a single MLD system exists with no legacy devices (STAs). However, with coexisting legacy devices, the throughputs under Sync and Sync-PL are greatly reduced. Sync fails to provide TXOPs on either link and shows a throughput lower than that in the legacy-only case. Under Sync, the existence of one link occupied by transmission involving other devices can severely affect performance because it can cause the Sync process to become stuck in the step of repicking the backoff counter because of busy channels. Sync-PL achieves performance close to the legacy-only case since its TXOPs are limited by the contention on the primary link. However, the other access schemes still show higher throughput than the legacy-only case, implying that MLO can significantly increase throughput performance. Async without power leakage always achieves double throughput due to the independent transmissions on both links. Sync-FT shows similar performance as Async because of its ability to allow fast transmission when a multi-link TXOP is available. While the other variants of Sync-FT show similar performance to each other, Sync-FT-Repick + Comp shows decreasing throughput as the number of legacy STAs increases.

Figure 18b shows the throughput performance without the STR capability. The extent to which Sync-FT suffers from power leakage is limited, and it becomes the best scheme. The variants of Sync-FT show similar performance trends as in the case with the STR capability. Async suffers from power leakage and achieves limited throughput, especially in the case of an EHT system alone. This means that Async fails to fully exploit the benefits of MLO with power leakage. As the number of legacy STAs increases, the performance gap between Async and Sync-FT decreases, showing that its throughput is predominantly limited by contention in this case. As in the case without power leakage, Sync and Sync-PL still suffer in the presence of legacy STAs.

#### 5.1.2. Coexistence with Legacy Devices

**Figure 19:**
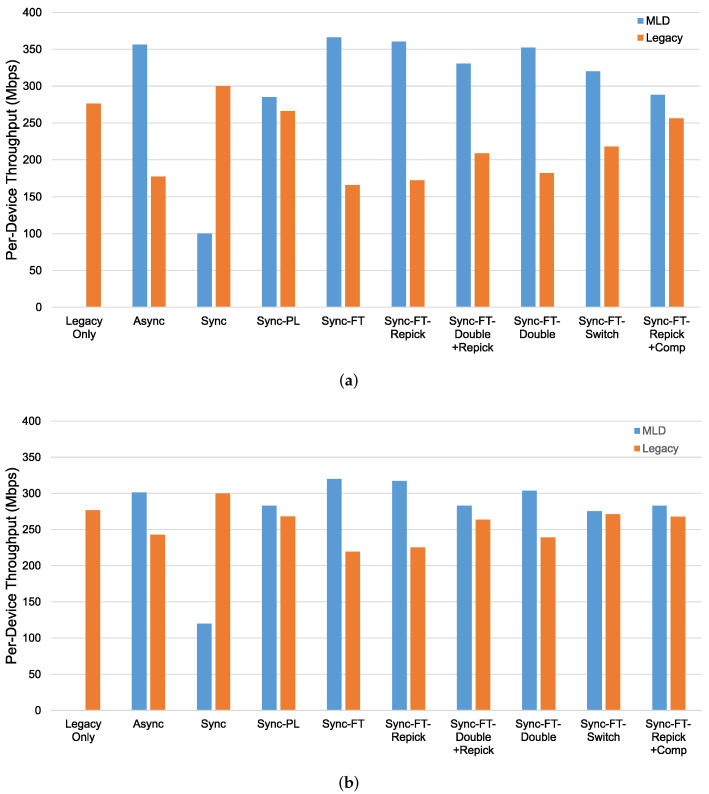
Throughput in the coexistence scenario (Phase 1 vs. 2) with single-spot deployment. (**a**) With STR capability, (**b**) Without STR capability.

Next, we investigate the coexistence performance of MLO. The throughput results for the coexistence evaluation scenario in Figure 17 with and without the STR capability are shown in Figure 19. We consider a network in which four devices coexist. The throughput for the legacy-only case, in which only legacy devices exist, is shown as a baseline. In the other cases, two legacy devices, one on each channel, are replaced with two MLDs, and the per-device throughput performance is shown for both the MLDs (blue bars) and the remaining legacy devices (orange bars). If the orange bar for a particular scheme is shorter than that for the legacy-only case, this means that under this scheme, the MLDs aggressively occupy the channel resources and fail to coexist with the legacy devices in a harmonious manner.

**Figure 20:**
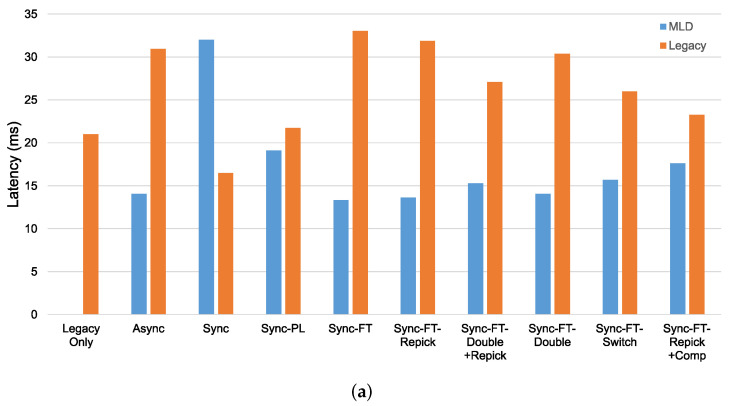

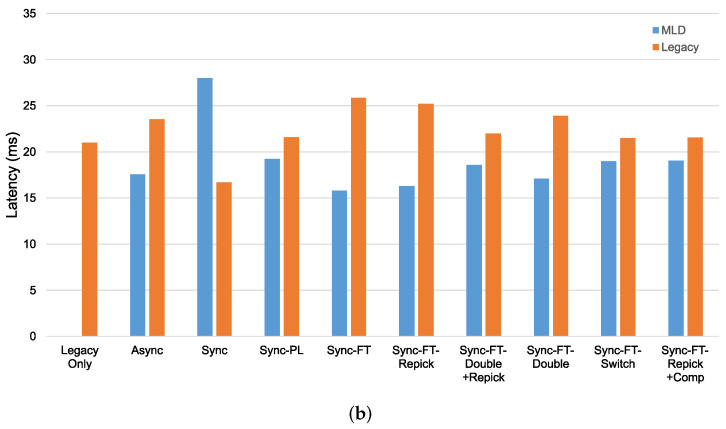
Latency in the coexistence scenario (Phase 1 vs. 2) with single-spot deployment. (**a**) With STR capability, (**b**) Without STR capability.

In the case with the STR capability, as shown in Figure 19a, we can see that most schemes except Sync, Sync-PL, and Sync-FT-Repick + Comp show significantly lower throughput of the legacy devices, implying that their throughput gains result from the decreased channel occupation time of the legacy devices. Sync shows significant performance degradation of the MLDs, while Sync-PL achieves a throughput similar to that with legacy devices only, as also shown in Figure 18. It is seen that Sync-FT performs aggressive operations, and its variants somewhat mitigate such aggressiveness. Among the variants, Sync-FT-Repick + Comp shows the throughput of the legacy devices that is closest to that in the legacy-only case, but its throughput gain is simultaneously reduced. Figure 19b shows the performance results without the STR capability. Most schemes result in a lower throughput of the MLDs than in the case with the STR capability, and thus, the legacy devices achieve more throughput, leading to smaller throughput gaps between the MLDs and legacy devices. The corresponding latency results are presented in Figure 20, showing that MLO is also beneficial in terms of latency. The observed coexistence performance trends in terms of latency are similar to the throughput trends described above.

**Figure 21:**
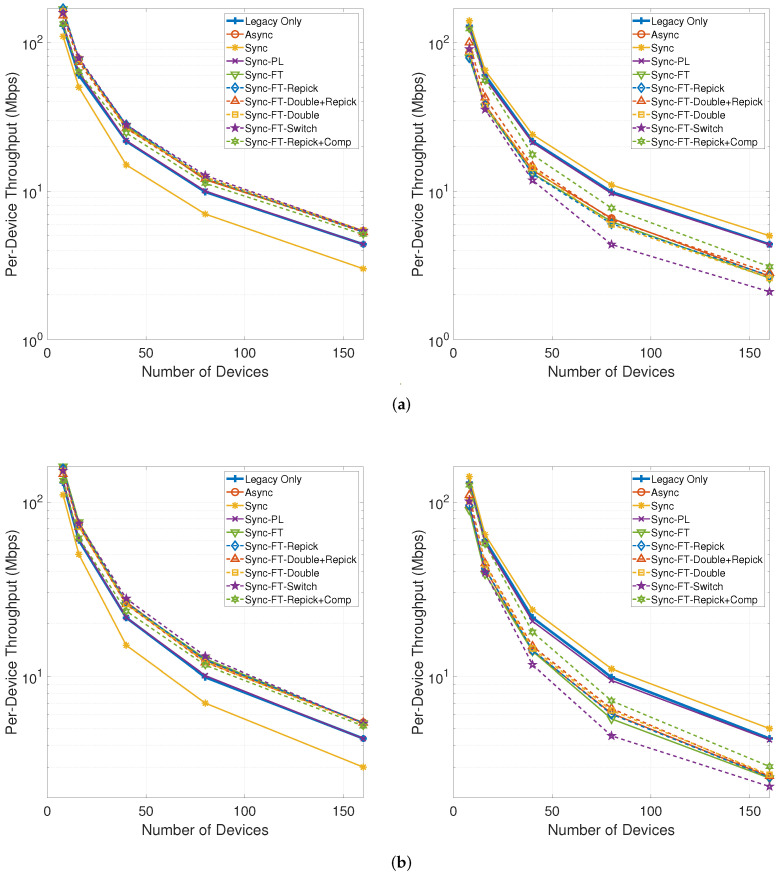
Throughput with an increasing number of devices in the single-spot deployment scenario (left: MLDs, right: legacy devices). (**a**) With STR capability, (**b**) Without STR capability.

Figure 21 shows the per-device throughput performance for an increasing number of total devices in the network. The performance trends are similar between the cases with and without the STR capability. Sync-PL achieves almost the same performance as the legacy-only case. Sync shows poorer performance than the legacy-only case, and its performance degradation increases as the number of devices increases. The other MLO schemes achieve better performance than the legacy-only case. The throughput performance of the legacy devices is exactly the same as for the legacy-only case when the legacy devices are coexisting with MLDs under Sync-PL but worsens with the other schemes. Moreover, the degradation for the legacy devices increases as the number of devices increases. Among the various schemes, Sync-FT-Repick + Comp is shown to achieve the best coexistence performance, although it offers poorer coexistence for a higher number of devices since the contention between MLDs is already high and the legacy devices receive fewer channel access opportunities.

#### 5.1.3. New Metric

To evaluate both the throughput and latency gains and the coexistence performance between different schemes using a single metric, we introduce a new metric γ. The goal of MLO is to gain as much throughput and latency performance as possible while maintaining acceptable coexistence with legacy devices/systems. Therefore, the MLD performance in an environment where legacy devices are present is compared against the performance of legacy STAs without any MLDs. The constraint itself comes from the difference in the legacy device performance before and after the addition of MLDs into the environment, and it is limited to zero (no positive values are allowed) because we only wish to leave the legacy performance unchanged rather than to boost the performance of the legacy devices. Let $\mu_{MLD,P2}$ be the throughput of an MLD in Phase 2, and let $\mu_{Leg,P1}$ and $\mu_{Leg,P2}$ be the throughputs of a legacy SLD in Phases 1 and 2, respectively. Furthermore, let $l_{MLD,P2}$, $l_{Leg,P1}$ and $l_{Leg,P2}$ be the corresponding packet latency values.

The new metrics, denoted by $\gamma_{t}$ for throughput and $\gamma_{l}$ for latency, are given as follows:

(1) $$\begin{array}{c} \gamma_{t}=\frac{\mu_{MLD,P2}}{\mu_{Leg,P1}}+\alpha \times \min\left\{1,\frac{\mu_{Leg,P2}}{\mu_{Leg,P1}}\right\}\\ \gamma_{l}=\frac{l_{Leg,P1}}{l_{MLD,P2}}+\alpha \times \min\left\{1,\frac{l_{Leg,P1}}{l_{Leg,P2}}\right\} \end{array}$$

where the first terms, $\mu_{MLD}/\mu_{Leg,P1}$ and $l_{Leg,P1}/l_{MLD}$, measure the performance gain of MLO and the second terms measure the coexistence performance. α is a weighting constraint; a larger α places more weight on the aspect of the coexistence of MLO with legacy systems. Since increasing the performance of legacy systems to higher than it was before is not a focus of MLO development, we upper-limit the second term by one. For each term, higher values are better. Therefore, higher $\gamma_{t}$ and $\gamma_{l}$ values imply better performance overall.

**Figure 22:**
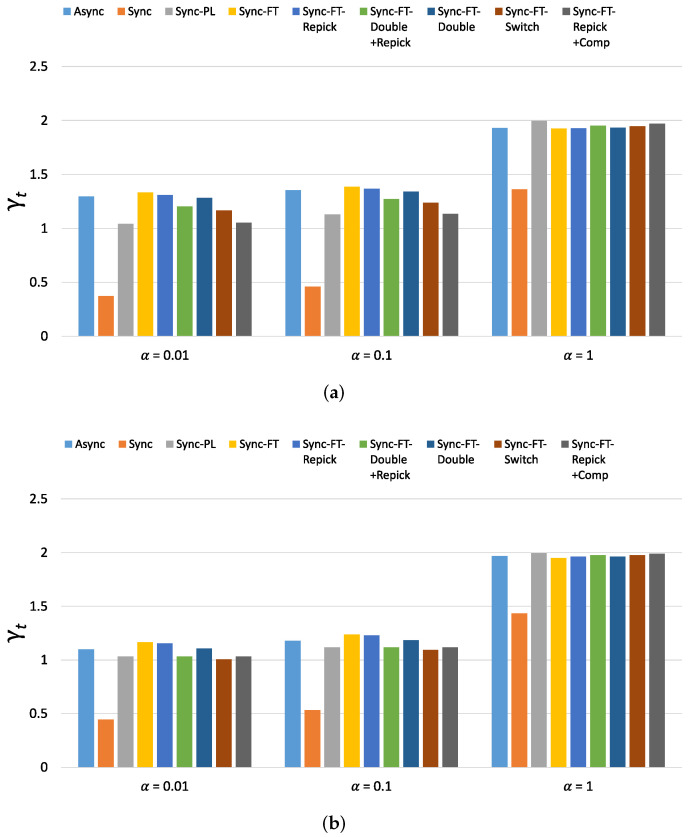
Values of the new metric $\gamma_{t}$ in the single-spot deployment scenario. (**a**) With STR capability, (**b**) Without STR capability.

The new metric of throughput performance, i.e., $\gamma_{t}$, is shown in Figure 22 for the considered schemes with various α values. (The new metric of latency performance, i.e., $\gamma_{l}$, shows similar values and trends as $\gamma_{t}$ and thus is omitted). When α=0.01, Async, Sync-FT and Sync-FT-Repick show the highest values, while Sync shows the lowest value. The other coexistence solutions show lower values than Sync-FT since they assign penalties to the MLDs. As α increases, the values of $\gamma_{t}$ for Sync, Sync-PL and the other coexistence solutions increase. When α=1, most schemes except Sync show similar values. This implies that the performance gain of MLO originates from a greater amount of channel access time and thus comes at the expense of performance degradation for legacy devices. Therefore, in general, the higher the performance gain of MLO is, the lower its coexistence performance.

### 5.2. Indoor Random Deployment Scenario

In this simulation, the deployment scenario follows the 3GPP’s indoor coexistence evaluation scenario [22], as shown in Figure 16. Each of two operators deploys four WLANs in a single-floor apartment building. Devices are evenly allocated to the WLANs. The devices of each WLAN are randomly distributed around its serving AP. In Phase 1, all APs are legacy APs (i.e., AP SLDs) working in different primary channels as shown in the figure, while in Phase 2, the APs function as AP MLDs that can work on both links, reserving their primary links only for Sync-PL operation. The system parameters follow those for the simulation scenario [23] and evaluation methodology [24] used for IEEE 802.11ax. The indoor path loss model of the IEEE 802.11ax simulation scenario is used. We consider the transmission bit rates of IEEE 802.11ac, which range from 6.5 (MCS 0) to 78 (MCS 9) Mbps. Packet errors are generated based on the packet error rate (PER) curves provided in the evaluation methodology for IEEE 802.11ax. The MCS of a link is taken to be the highest bit rate with a PER lower than 10% at the signal-to-noise ratio (SNR) of the link [25,26]. Acknowledgment frames are transmitted at the lowest transmission bit rate (6.5 Mbps). The noise figure is 7 dB, and the noise floor is −94 dBm. The simulation parameters are listed in Table 1.

**Figure 23:**
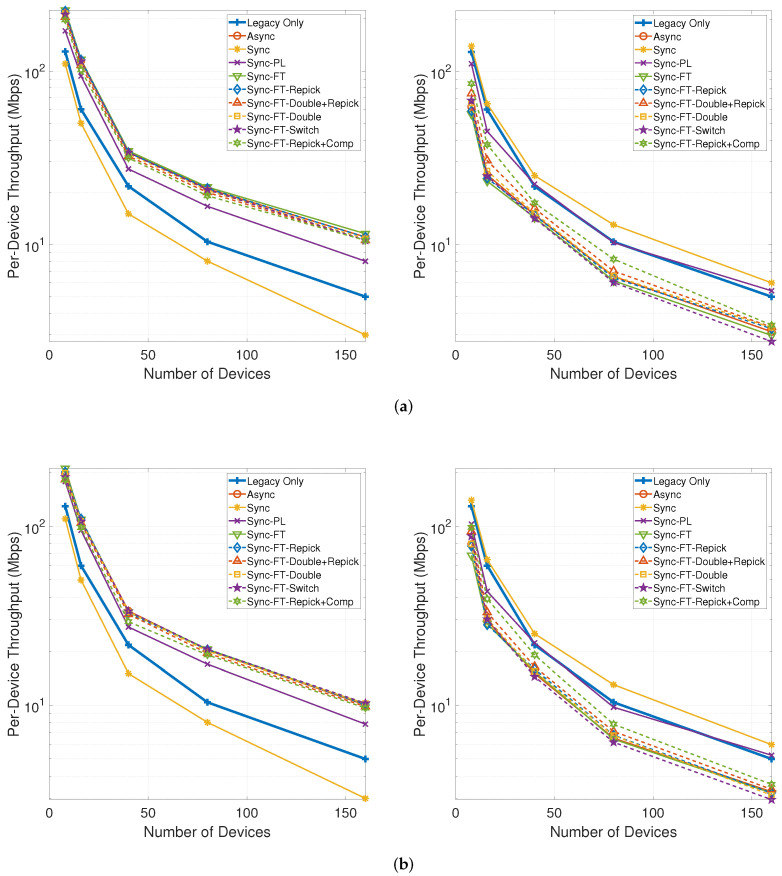
Throughput with an increasing number of devices in the indoor random deployment scenario (left: MLDs, right: legacy devices). (**a**) With STR capability, (**b**) Without STR capability.

**Figure 24:**
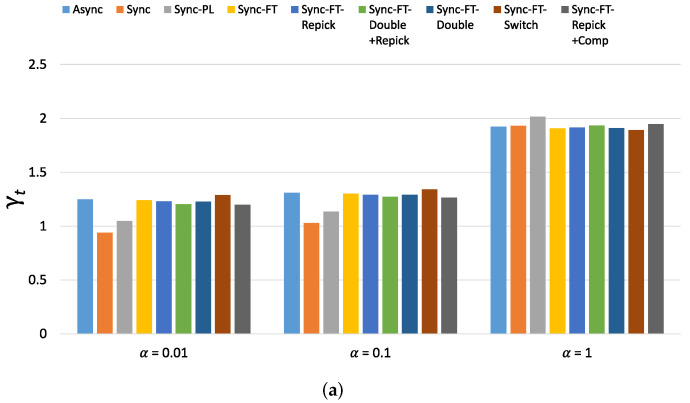

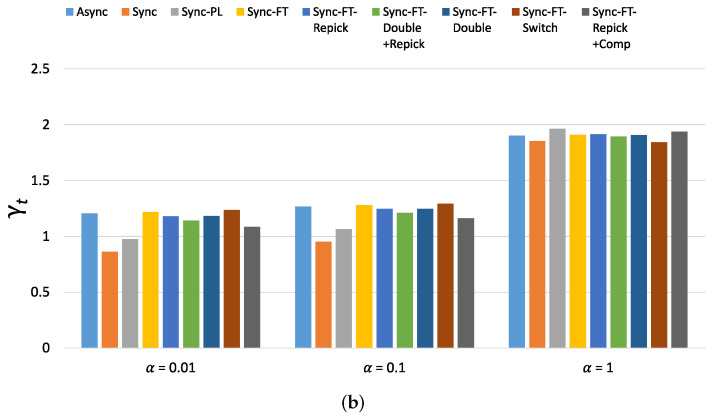
Values of the new metric $\gamma_{t}$ for 80 devices in total in the indoor random deployment scenario. (**a**) With STR capability, (**b**) Without STR capability.

Figure 23 shows the per-device throughput performance for an increasing number of total devices. The overall trends are similar to those observed in the single-spot deployment scenario. Sync-FT and its coexistence solutions achieve better performance than the legacy-only case. Sync-PL shows almost the same throughput performance as that achieved with legacy devices only. As the number of devices increases, the throughput gain of Sync-FT also increases. In contrast, the performance degradation of the legacy devices is more severe than in the single-spot deployment scenario. Sync-FT-Repick+Comp still shows the best coexistence performance among the proposed solutions. Similar trends of $\gamma_{t}$ are also observed in Figure 24.

## 6. Conclusions

In this paper, we illustrated the MLO functionality of the upcoming IEEE 802.11be standard and described how the channel access schemes of IEEE 802.11be facilitates multi-link utilization. We demonstrated the coexistence challenge presented by the enhanced synchronous channel access scheme, and we proposed solutions to mitigate this challenge. Through comprehensive simulations, we evaluated the throughput and latency gains of MLO and the proposed solutions relative to legacy systems in various network environments. At the same time, we investigated the coexistence performance of MLO with and without the STR capability and demonstrated that the proposed solutions mitigate the coexistence problem of the enhanced synchronous channel access scheme to different degrees. A metric for evaluating both the throughput and latency gains and the coexistence performance of a multi-link channel access scheme using a single value was also proposed. The evaluation results showed that, among the proposed solutions, compensating the backoff count achieves the best coexistence performance with a marginal throughput decrease of MLDs. However, there may arise a backoff count overflow problem that the backoff count of an MLD accumulated by repeated compensations gets excessive, thus not giving a transmission opportunity to the MLD once an overflow occurs. We leave an extended design of the compensation feature to handle this problem as future work. As machine learning has been applied to a wide range of communication and networking problems [27], developing a machine learning-based channel access scheme for MLO is also a subject for future research.

## Figures and Tables

**Table 1:** Feature combinations considered in the proposed solutions.

	Solution	Repick	Switch	Double + Repick	Double	Repick + Comp
Feature						
Repicking a backoff count		✓		✓		✓
Doubling CW				✓	✓	
Switching to another CW			✓			
Backoff count compensation						✓

**Table 2:** Simulation parameters.

Parameter	Value
Frequency	5 GHz
Number of links per MLD	2
Channel bandwidth of a link	80 MHz
Total number of devices	Single-spot: 1–11, 4, [8, 16, 40, 80, 160]
	Indoor random: [8, 16, 40, 80, 160]
MCS	Single-spot: 7 (680.6 Mbps)
	Indoor random: Adaptation (0∼9)
Traffic generation	Full buffer
Max aggregation size	64 MPDUs
MPDU size	1500 bytes
Slot length	9 μs
SIFS	16 μs
DIFS	34 μs
CWmin	16 (32 for set B)
CWmax	1024 (2048 for set B)
Tx power	18 dBm for both AP and user device
Energy detection threshold	−62 dBm
Preamble detection threshold	−82 dBm

## Data Availability

Not applicable.

## Abbreviations

The following abbreviations are used in this manuscript:

AP	Access Point
BO	BackOff procedure
BSS	Basic Service Set (BSS)
CCA	Clear Channel Assessment
CW	Contention Window
CWmax	Contention Window maximum
CWmin	Contention Window minimum
DIFS	Distributed coordination function InterFrame Space
ED	Energy Detection
EHT	Extremely High Throughput
FT	Faster Transmission
LBT	Listen-Before-Talk scheme
LLC	Logical Link Control layer
LMAC	Lower-Medium Access Control
MAC	Medium Access Control
MCS	Modulation and Coding Scheme
MLO	MultiLink Operation
MLD	MultiLink Device
MU-EDCA	MultiUser Enhanced Distributed Channel Access
OBSS	Overlapping Basic Service Set
PHY	PHYsical layer
PIFS	Point coordination function InterFrame Space
QoS	Quality of Service
RF	Radio Frequency
SAP	Service Access Point
STR	Simultaneous Transmission and Reception
SLD	Single-Link Device
SLO	Single-Link Operation
TCP	Transmission Control Protocol
TID	Traffic IDentifier
UMAC	Upper-Medium Access Control
WLAN	Wireless Local Area Network
